# Iterative integral parameter identification of a respiratory mechanics model

**DOI:** 10.1186/1475-925X-11-38

**Published:** 2012-07-18

**Authors:** Christoph Schranz, Paul D Docherty, Yeong Shiong Chiew, Knut Möller, J Geoffrey Chase

**Affiliations:** 1 Institute of Technical Medicine, Furtwangen University, Jakob-Kienzle-Str. 17, Villingen-Schwenningen, Germany; 2 Department of Mechanical Engineering, University of Canterbury, Private Bag 8140, Christchurch, New Zealand

**Keywords:** Parameter identification, Respiratory mechanics, Viscoelastic model, Global minimum, Robustness

## Abstract

**Background:**

Patient-specific respiratory mechanics models can support the evaluation of optimal lung protective ventilator settings during ventilation therapy. Clinical application requires that the individual’s model parameter values must be identified with information available at the bedside. Multiple linear regression or gradient-based parameter identification methods are highly sensitive to noise and initial parameter estimates. Thus, they are difficult to apply at the bedside to support therapeutic decisions.

**Methods:**

An iterative integral parameter identification method is applied to a second order respiratory mechanics model. The method is compared to the commonly used regression methods and error-mapping approaches using simulated and clinical data. The clinical potential of the method was evaluated on data from 13 Acute Respiratory Distress Syndrome (ARDS) patients.

**Results:**

The iterative integral method converged to error minima 350 times faster than the Simplex Search Method using simulation data sets and 50 times faster using clinical data sets. Established regression methods reported erroneous results due to sensitivity to noise. In contrast, the iterative integral method was effective independent of initial parameter estimations, and converged successfully in each case tested.

**Conclusion:**

These investigations reveal that the iterative integral method is beneficial with respect to computing time, operator independence and robustness, and thus applicable at the bedside for this clinical application.

## Introduction

Mechanical ventilation (MV) therapy in intensive care units (ICU) carries the risk of severe additional complications for the patient due to non-optimal ventilator settings [1]. To reduce patient risk, optimal patient-specific ventilator settings must be found. Mathematical models of respiratory mechanics effectively predict the outcome of specific ventilator settings and thus support clinical evaluation of lung protective settings [2]. Prediction quality depends on how well model parameters correspond to the true patient properties and on the accuracy of the model representing the individual’s lung physiology. To assure clinical applicability at the bedside, especially in closed loop settings [2,3], reliable and immediate parameter identification is required. However, information for analyzing respiratory mechanics directly at the bedside is generally restricted to measurements of airway pressure and flow rate. Therefore, any model must enable easy and fast parameterization in terms of the available clinical data. Thus, models should capture all necessary dynamics and be as simple as possible. Hence, lumped parameter models are a common approach for optimizing ventilator settings [4].

Typical parameter identification methods minimize the least square error (LSE) between measured samples and model simulations. For single compartment models of respiratory mechanics, multiple linear regression is an established straightforward approach [5,6]. Multiple linear regression is also applicable to higher order linear models, but the quality of results can be distorted by noise [6]. Alternative parameter identification methods for higher-order and nonlinear models are iterative error-mapping methods [7-11], such as the Simplex-Search Method [12] or Levenberg-Marquardt Algorithm [13,14]. These methods require initial guesses for the model parameters and iterate towards LSE values by approaching the minimum on the error surface. However, with an increasing number of degrees of freedom, several parameter constellations can appear as additional possible solutions (local minima) [8]. Hence, such methods can report non-optimal parameter values and are highly dependent on initial parameter estimates. To reduce the probability of convergence to local minima, various global search strategies, such as random search, simulated annealing or genetic algorithms can be used [15]. However, these methods are time consuming and the quality of the solution is sensitive to the parameterization of the algorithm [16].

This research presents an iterative integral method (IIM) [17,18] for respiratory mechanics parameter identification, and compares the performance against established parameter identification methods. The method was originally developed for parameter identification of glucose-insulin models [19]. In the context of the glucose-insulin models, the IIM is comparatively simple to apply, requires comparatively minimal computing time and does not require the estimation of initial values [17,19]. The IIM is tested with simulation data to demonstrate its robustness and efficiency, and clinical study data is used to demonstrate the clinical potential.

## Model & methodology

### Viscoelastic model

Viscoelastic Models (VEM) of respiratory mechanics are established models and assume that the tissues comprising the walls of the alveolar compartment are viscoelastic, rather than simply elastic [20]. The VEM used in this research is an established model of respiratory mechanics whose applicability with respect to healthy and lungs with Acute Respiratory Distress Syndrome (ARDS) has been demonstrated [21,22]. Ganzert et al. [10] showed the different trends of the VEM parameters in healthy and ARDS subjects, which might be used as indicator for ARDS development.

**Figure 1:**
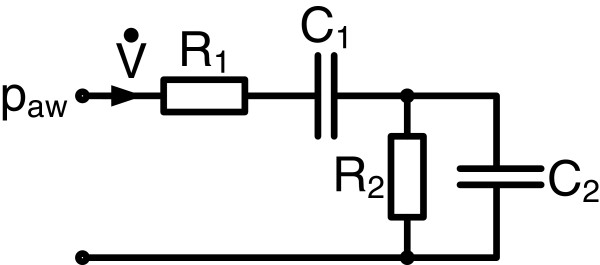
**Viscoelastic Model of Respiratory Mechanics.***R*_*1*_ denotes the airway resistances and *C*_*1*_ the static compliance of the respiratory system. *R*_*2*_ and *C*_*2*_ are the resistance and the compliance of the viscoelastic component. The respiratory airflow $\dot{V}$ represents the input and the airway pressure *p*_*aw*_ the output of the model.

The analogous electrical circuit for the VEM is shown in Figure 1 and the mathematical description is presented in state-space representation in Eq. (1):

(1) $$\begin{array}{l} \left[\begin{array}{c} {\dot{p}}_{C1}\\ {\dot{p}}_{C2} \end{array}\right]=\left[\begin{array}{cc} 0 & 0\\ 0 & -1/\left(R_{2}C_{2}\right) \end{array}\right]\left[\begin{array}{c} p_{C1}\\ p_{C2} \end{array}\right]+\left[\begin{array}{c} 1/C_{1}\\ 1/C_{2} \end{array}\right]\dot{V}\\ p_{\mathrm{aw}}=\left[\begin{array}{cc} 1 & 1 \end{array}\right]\left[\begin{array}{c} p_{C1}\\ p_{C2} \end{array}\right]+R_{1}\dot{V} \end{array}$$

The flow rate $\dot{V}$ [mL/s] defines the model input and the airway pressure *p*_aw_ [cmH_2_O] defines the model output. Airway resistance is described by *R*_1_ [cmH_2_O·s/mL] and the static lung compliance by *C*_*1*_ [mL/cmH_2_O]. *R*_*2*_ [cmH_2_O·s/mL] and *C*_2_ [mL/cmH_2_O] account for the viscoelasticity described by the viscoelastic time constant *R*_*2*_·*C*_*2*_. *p*_*C1*_ and *p*_*C2*_ [cmH_2_O] are the internal state variables being the pressure in the compliant static and viscoelastic lung compartment.

The four parameters (*R*_1_, *C*_*1*_, *R*_2_, *C*_2_) correspond to the unknown patient-specific parameters:

(2) $$X_{\mathrm{VEM}}:=\left\{R_{1},C_{1},R_{2},C_{2}\right\}$$

A fundamental prerequisite for successful parameter identification is *a-priori* structural identifiability of the parametric model [23]. This necessary criterion states that under ideal conditions of noise-free observations and error-free model structure, the unknown parameters of the model can be uniquely recovered from the measured input–output variables. The VEM is globally identifiable [8] using the DAISY (Differential Algebra for Identifiability Systems) computer program to automatically check for *a-priori* structural identifiability [23].

### Data

First, the parameter identification methods were compared *in-silico* using synthetic data sets generated by simulating parameterized VEMs. Second, the methods were tested using clinical data to assess their clinical applicability.

#### Simulation data

A flow profile corresponding to the Volume-Controlled-Ventilation (VCV) mode was applied to parameterized VEMs. Inflation was simulated by a constant flow of 500 mL/s over 1 s, followed by an end-inspiratory pause (EIP) of 4 s corresponding to the closure of the expiratory valve as observed in clinical data [24].

100 different sets of model parameters were generated for a Monte-Carlo Analysis. The model parameters were randomly selected from an evenly distributed range between 0.5 and 1.5 times a realistic physiological parameter set (*R*_*1*_ = 0.010 cmH_2_O·s/mL, *C*_*1*_ = 30.00 mL/cmH_2_O, *R*_*2*_ = 0.020 cmH_2_O·s/mL, *C*_*2*_ = 80.00 mL/cmH_2_O).

**Figure 2:**
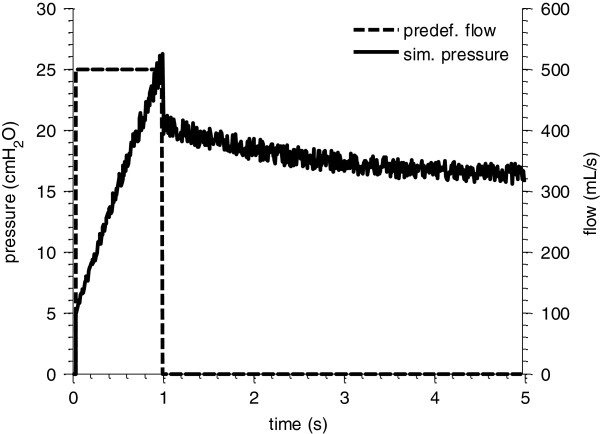
**Predefined flow profile and simulated pressure response of the VEM.** The simulation consists of an inspiration part (1 s), followed by an end-inspiratory pause (4 s).

After simulating each VEM, 10 different sets of white noise (5% of given pressure sample) were added in order to impose noisy measurement error. An example of the simulated pressure response is shown in Figure 2. It consists of an increase during inspiration and an exponential drop during the EIP.

#### Clinical data

Measurement sets from N = 13 mechanically ventilated patients were selected from a previous ARDS– Study [10,25], where SCASS-Maneuvers (Static Compliance Automated Single Step) were performed. The SCASS-Maneuver consists of airway occlusions within the inspiration phase of a breathing cycle that are initiated when a randomized inspiration volume is reached. The measurement set consisted of flow rate ($\dot{V}$) and airway opening pressure (*p*_*aw*_) signals sampled at 125 Hz. For each patient, two breathing cycles were extracted. During occlusion, the airway pressure reached a quasi-static equilibrium exponentially.

The study was approved by the local ethics committees of the participating university hospitals. Informed consent was obtained from patients or their legally authorized representative. The detailed description of the experimental setup is shown in [25].

### Parameter identification

Five parameter identification methods were used to identify the patient-specific VEM parameters Eq. (2) using the simulation and clinical data. These methods also provide references for the iterative integral method. To compare computational efficiency, all methods were tested on the same Desktop PC (Intel Core 2 Duo, 2.80 GHz), where computation time was measured.

#### Multiple linear regression (MLR)

Given a linear model, MLR is an obvious approach for parameter identification [5,6]. MLR requires the input–output relation of the model, eliminating the state variables. This relation is derived from the state space description of the VEM by calculating the derivative of the output equation from Eq. (1) and inserting the definition of the state variables:

(3) $${\dot{p}}_{\mathrm{aw}}={\dot{p}}_{C1}+{\dot{p}}_{C2}+R_{1}\ddot{V}=\frac{1}{C_{1}}\dot{V}-\frac{1}{R_{2}C_{2}}p_{C2}+\frac{1}{C_{2}}\dot{V}+R_{1}\ddot{V}$$

To eliminate *p*_*C2*_ in Eq. (3), the output of Eq. (1) is rewritten in terms of *p*_*C2*_:

(4) $$p_{C2}=p_{\mathrm{aw}}-p_{C1}-R_{1}\dot{V}=p_{\mathrm{aw}}-\frac{1}{C_{1}}V-R_{1}\dot{V}$$

Eq. (4) is inserted in Eq. (3) leading to the input–output relation of the model:

(5) $${\dot{p}}_{\mathrm{aw}}=-\frac{1}{R_{2}C_{2}}p_{\mathrm{aw}}+R_{1}\ddot{V}+\left(\frac{1}{C_{1}}+\frac{1}{C_{2}}+\frac{R_{1}}{R_{2}C_{2}}\right)\dot{V}+\frac{1}{R_{2}C_{1}C_{2}}V$$

Effectively, Eq. (6) re-arranges the state-space equation Eq. (1) to place the model variables as functions of measured values (*p*_*aw*_ and $\dot{V}$) and their derivatives or integrals.

(6) $$p_{\mathrm{aw}}=-R_{2}C_{2}{\dot{p}}_{\mathrm{aw}}+R_{1}R_{2}C_{2}\ddot{V}+\left(\frac{R_{2}C_{2}+R_{2}C_{1}+R_{1}C_{1}}{C_{1}}\right)\dot{V}+\frac{1}{C_{1}}V$$

MLR requires a sum of independent variables scaled by a multiplicative factor. Therefore the coefficients of Eq. (6) can be represented by new variables:

(7) $$p_{\mathrm{aw}}=A{\dot{p}}_{\mathrm{aw}}+B\ddot{V}+C\ddot{V}+DV$$

For parameter identification according to the Least-Square-Error (LSE) principle, Eq. (7) was arranged in matrix form:

(8) $$\begin{array}{ll} \left[\begin{array}{cccc} {\dot{p}}_{\mathrm{aw}}(1) & \ddot{V}(1) & \dot{V}(1) & V(1)\\ {\dot{p}}_{\mathrm{aw}}(2) & \ddot{V}(2) & \dot{V}(2) & V(2)\\ {\dot{p}}_{\mathrm{aw}}(3) & \ddot{V}(N) & \dot{V}(N) & V(N) \end{array}\right] & \left[\begin{array}{c} A\\ B\\ C\\ D \end{array}\right] \end{array}=\left[\begin{array}{c} p_{\mathrm{aw}}(1)\\ p_{\mathrm{aw}}(2)\\ p_{\mathrm{aw}}(N) \end{array}\right]$$

Values of *A-D* are identified applying the Moore-Penrose pseudo-division for over-defined matrix equations. Patient-specific *R*_*1*_*, C*_*1*_*, R*_*2*_*, C*_*2*_ values are then uniquely derived from the identified values of *A-D*.

#### Integral Method (IM)

The IM is similar to MLR in terms of identification steps. However, integrals are used to improve robustness to noise [19]. Hence, Eq. (5) was integrated assuming *p*_*aw*_(0) = 0:

(9) $$p_{\mathrm{aw}}=-\frac{1}{R_{2}C_{2}}\int p_{\mathrm{aw}}dt+R_{1}\dot{V}+\left(\frac{1}{C_{1}}+\frac{1}{C_{2}}+\frac{R_{1}}{R_{2}C_{2}}\right)V+\frac{1}{R_{2}C_{1}C_{2}}\int Vdt$$

The coefficients of Eq. (9) are represented by new variables:

(10) $$A=-\frac{1}{R_{2}C_{2}}$$

(11) $$B=R_{1}$$

(12) $$C=\frac{1}{C_{1}}+\frac{1}{C_{2}}+\frac{R_{1}}{R_{2}C_{2}}$$

(13) $$D=\frac{1}{R_{2}C_{1}C_{2}}$$

Yielding:

(14) $$p_{\mathrm{aw}}=A\int p_{\mathrm{aw}}dt+B\dot{V}+CV+D\int Vdt$$

Incorporating Eq. (11) into an over-defined matrix system yields:

(15) $$\left[\begin{array}{cccc} \int_{0}^{1}p_{\mathrm{aw}}dt & \dot{V}(1) & V(1) & \int_{0}^{1}Vdt\\ \int_{0}^{2}p_{\mathrm{aw}}dt & \dot{V}(2) & V(2) & \int_{0}^{2}Vdt\\ \int_{0}^{N}p_{\mathrm{aw}}dt & \dot{V}(N) & V(N) & \int_{0}^{N}Vdt \end{array}\right]\left[\begin{array}{c} A\\ B\\ C\\ D \end{array}\right]=\left[\begin{array}{c} p_{\mathrm{aw}},(1)\\ p_{\mathrm{aw}}(2)\\ p_{\mathrm{aw}}(N) \end{array}\right]$$

Patient-specific parameters *R*_*1*_*, C*_*1*_*, R*_*2*_*, C*_*2*_ are uniquely regained using the identified values of *A-D:*

(16) $$R_{1}=B$$

(17) $$R_{2}C_{2}=-\frac{1}{A}$$

(18) $$C_{1}=\frac{1}{DR_{2}C_{2}}$$

(19) $$C_{2}=\frac{R_{2}C_{1}C_{2}}{CR_{2}C_{1}C_{2}-R_{2}C_{2}-R_{1}C_{1}}$$

#### Iterative Integral Method (IIM)

The IIM uses much the same method as the IM. However, the identified *A-D* values are used to re-simulate *p*_*aw*_ according to Eq. (11). Resimulated *p*_*aw*_ is then used to update the left-hand-side (LHS) integrals of Eq. (12). Subsequently, Eq. (12) is solved again yielding updated values of *A-D*. This process is repeated until convergence. The sum of squared error (SSE) between simulated and measured *p*_*aw*_ was calculated after every iteration. The relative termination tolerance (TolFUN) for changes in the function value (SSE) was set to 10^-4^ This value is in accordance to the default setting of the proprietary gradient-based search methods in MATLAB.

#### Proprietary Levenberg-Marquardt Algorithm (LMA) and Simplex Search Method (SSM)

Gradient-Based Methods such as the Levenberg-Marquardt Algorithm (LMA) (MALTAB command: lsqnonlin) and the Simplex-Search Method (SSM) (MALTAB command: fminsearch) were applied as a references to the IIM using following default convergence criteria: terminate the optimization if the relative change in parameters is smaller than 10^-4^ (MATLAB: TolX value) or when the relative change in the SSE value is smaller than 10^-4^ (MATLAB: TolFUN value).

Gradient-Based Methods require initial estimates for the corresponding parameters. Initial estimates close to the global minimum increase the probability of successful parameter identification.

For the case of identifying simulated data, the parameter values that were used for simulation are known and were used as initial values to provide the fastest, easiest solution, and a conservative comparison.

**Figure 3:**
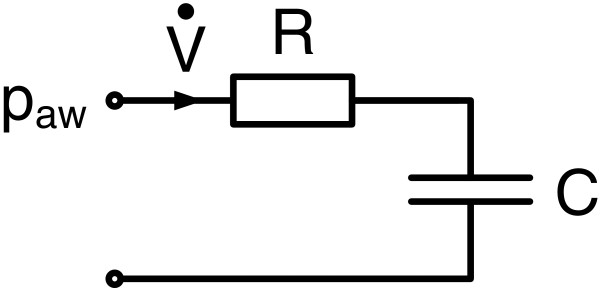
**1**^**st**^**Order Model (FOM).***R* denotes the airway resistance and *C* static compliance of the respiratory system. The respiratory airflow $\dot{V}$ represents the input and the airway pressure *p*_*aw*_ the output of the model.

For the case of clinical data, patient outcomes are initially unknown. Thus, convenient estimates for initial values are important. To decrease the potentially deleterious influence of poorly estimated initial values for gradient-based methods, a hierarchical method was developed by Schranz et al. [8]. This approach uses identification of simple respiratory models to provide prior knowledge. It is preferable for the simpler models to be identified with linear regression to avoid the influence of initial values. The identified parameter values are then used as initial values to support the identification of more complex models. In the VEM case, the 1^st^ order model (FOM) of respiratory mechanics (Figure 3) and Equation (14) was first identified by MLR leading to estimates of airway resistance (*R*) and respiratory compliance (*C*).

(20) $$p_{\mathrm{aw}}=R\dot{V}+\frac{1}{C}\int Vdt$$

Additionally, the time constant τ of the exponential pressure drop during the zero-flow phase (Figure 2) was estimated by extracting the corresponding pressure interval fitted by an exponential function. Given an error-free model structure, this estimated time constant corresponds to the viscoelastic time constant *R*_*2*_*·C*_*2*_. With the information of R, C and τ, initial values can be provided for gradient-based parameter identification of the VEM. This method improved the robustness of the subsequent parameter identification significantly by reducing the potential for poorly estimated initial values to result in local minima identification [8]. It is thus a more effective and conservative method comparison for the IIM.

## Results

### Parameter identification using simulation data

The identified parameter values from each parameter identification method were equal in the noise free synthetic data set and were effectively equal to the simulation values (R = 1.000). However, adding noise to the simulation data causes the true global minimum to slightly shift away from the original parameter values. In this case, the solutions reported by the SSM were used to define the new error minimum. The Monte-Carlo analysis revealed that the IIM, IM, SSM and LMA reported the same solution error minimum in every data set. The MLR didn’t locate a reasonable error minimum in any single case. The resulting parameter values of the MLR deviate by up to 100% from the values at the reported error minima. This outcome led to a median SSE 380% larger than the SSE of the error minima. The IIM always reached convergence after its first iteration and was thus equivalent to the IM in this case. The required median [interquartile] computing time of the IM and IIM was 0.8 [0.7-0.9] ms. In comparison, the gradient-based methods require 108.3 [101.6-115.7] ms for the SSM and 15.1 [15.0 – 15.5] ms for LMA.

### Parameter identification using clinical data

All results using the clinical data are summarized in Table 1. Within all clinical data sets, the SSM converged to a minimal SSE with physiologically plausible parameter values. These values are considered as error minimum. The SSM required an average of 251 ms per data set. The LMA found the same minima in 17 data sets, but located local non-physiological minima with partly negative parameter values and higher SSE values in 9 out of 26 (35%) data sets. MLR identification resulted in non-physiological parameter values and significantly higher SSE values in every (100%) data set. IM identification reported non-physiological parameter values that were either negative or significantly higher than the error minimum in 8 of 26 (31%) data sets with higher SSE values than the SSM. Parameter identification using the IIM resulted in similar minima as the SSM in every data set.

**Table 1:** Median and minimal-, maximal values of the identified parameters with SSE and required computing time

**Method**		***R***_***1***_	***C***_***1***_	***R***_***2***_	***C***_***2***_	***SSE***	***t***_Comp_^**^
		**[cmH**_**2**_**O·s/mL]**	**[mL /cmH**_**2**_**O]**	**[cmH**_**2**_**O·s/mL]**	**[mL /cmH**_**2**_**O]**	**[(cmH**_**2**_**O)**^**2**^**]**	**[ms]**
IIM	min	0.005	15.05	0.006	64.09	73.7	2.59
	**med**	**0.014**	**32.32**	**0.014**	**145.33**	**334.3**	**5.02**
	max	0.032	54.60	0.042	411.05	1 998.4	10.48
MLR	min	0.000	14.58	< 0.000*	> 10 000.00*	4 119.0	0.71
	**med**	**0.000**	**31.07**	< 0.000*	> 10 000.00*	**18 511.0**	**0.74**
	max	0.000	48.95	> 10.000*	> 10 000.00*	90 526.7	0.98
IM	min	0.005	15.17	0.003	< 0.00*	74.6	0.87
	**med**	**0.014**	**33.85**	**0.017**	**104.26**	**342.5**	**0.93**
	max	0.033	69.12	1.039*	177.61	2 066.3	1.42
SSM	min	0.005	15.07	0.006	64.05	73.7	204.65
	**med**	**0.014**	**32.35**	**0.015**	**142.39**	**334.3**	**251.10**
	max	0.032	55.14	0.042	398.30	1 997.2	350.96
LMA	min	0.007	14.98	< 0.000*	64.05	153.6	62.50
	**med**	**0.014**	**32.35**	**0.012**	**181.41**	**368.4**	**71.88**
	max	0.032	55.14	0.042	1 071.58	1 997.2	96.69

* value not within a physiologically plausible range, ** computing time.

Table 2 shows the IIM convergence in an example data set where the IM reported erroneous results. Starting from the erroneous IM result (0^th^ iteration), the SSE and non-physiological parameter values converge step by step towards the error minimum approaching physiologically plausible parameter values.

**Table 2:** Parameter convergence of the Iterative Integral Method (IIM) compared to the Simplex-Search Method (SSM)

**Method**	**Iteration Nr.**	***R***_***1***_	***C***_***1***_	***R***_***2***_	***C***_***2***_	***SSE***
		**[cmH**_**2**_**O·s/mL]**	**[mL /cmH**_**2**_**O]**	**[cmH**_**2**_**O·s/mL]**	**[mL /cmH**_**2**_**O]**	**[(cmH**_**2**_**O)**^**2**^**]**
IIM	0 (=IM)	0.021	44.91	0.003	−1 165.13*	1 486.25
	1	0.021	56.72	0.031	206.16	901.88
	2	0.021	54.73	0.027	238.23	853.05
	3	0.021	53.16	0.020	273.31	849.38
	4	0.021	52.77	0.019	284.32	849.19
	5	0.021	52.67	0.018	287.34	849.19
SSM	335	0.021	53.64	0.023	265.05	849.11

* value not within a physiologically plausible range.

**Figure 4:**
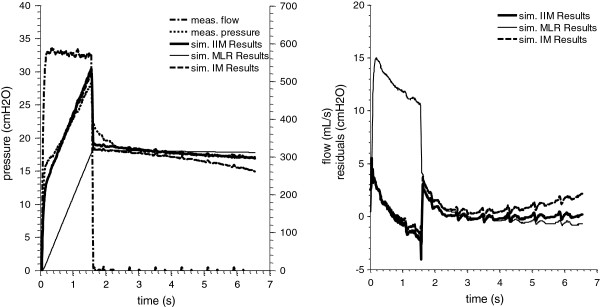
**Simulation Results.** Exemplary measurement set of pressure and flow with the simulated pressure responses and residuals of the VEM. VEM identified by the Iterative Integral Method (IIM), Multiple Linear Regression (MLR) and Integral Method (IM). Note, even if the IIM and the IM reported different parameters (Table 2), the curves are almost congruent.

The median required computing time in Table 1 for the IIM equals 5.02 ms reaching the minimum error faster than the SSM by a factor of 50. Example pressure responses for parameter sets identified by IIM, IM and MLR are shown together with the residuals in Figure 4. The residuals of the pressure response produced by IM parameter values are higher than the response of IIM parameter values. The pressure responses of the parameter sets identified by SSM and LMA are congruent with the solution of the IIM. Therefore these plots are neglected in Figure 4.

**Figure 5:**
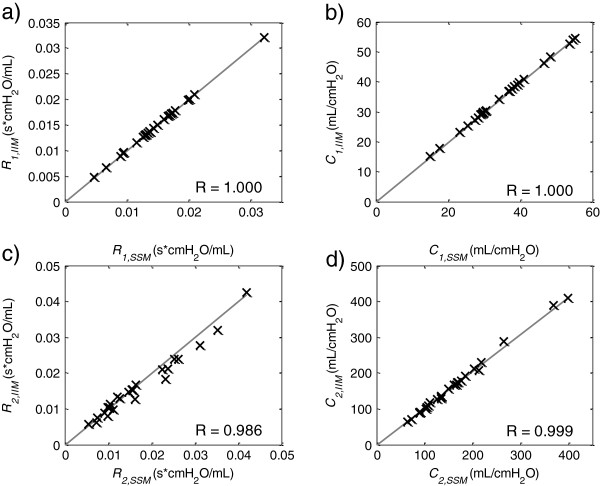
**Correlation Plots.** Correlation of the VEM parameter values identified by the Simplex-Search Method (SSM) and by the Iterative Integral Method (IIM) of 26 data sets.

Figure 5 shows the correlation plots of the reported IIM and SSM parameter values and confirms that the same solutions were reached in each data set. As the termination of the method is determined by convergence tolerances, minimal deviations occur in each parameter, leading to correlations lower than 1.000, ranging from R = 0.986 – 1.000. The correlation for the MLR, IM and LMA to the SSM are R = −0.209 –0.992, R = −0.448 – 0.999, R = 0.158 – 0.999, respectively.

## Discussion

The noise-free *in-silico* analysis showed that all identification methods where capable of identifying the parent parameter values via the simulated model response. This result confirms that the model is structurally identifiable [8,23], and the identification methods were accurately implemented. When limited white noise was added to the simulated profile all tested methods, except MLR successfully located a similar error minimum with identified parameter values close to their parent values. MLR resulted in significantly higher SSE values in every single data set and parameter values that were highly removed from their parent values. These erroneous results are caused by the noise-amplifying effects of derivative terms in Equation (6). The differentiation terms in Equation (6) are due to the second-order characteristics of the underlying two-compartment model. However, for some simpler models (e.g. linear and non-linear one-compartment models) MLR can be a reasonable and legitimate method for parameter identification [5,8]. However, this analysis indicates that MLR should not be applied for parameter identification of this VEM or such other higher-order models.

While the IM utilizes much the same methodology as the MLR, the outcomes of the noisy *in-silico* analysis and the clinical analysis show the merit of integrating the governing equation. The integrals in the IM low-pass filter the measured signals, yielding significant improvement in robustness over MLR for noisy simulation data. However, the IM located some non-physiological parameter values in the clinical data. In total, there were negative parameter values in 8 of 26 (31%) data sets. The erroneous parameter constellations resulted in higher residuals than reported by SSM and IIM methods, but still produced reasonable simulation results. These unacceptable parameter values results are in accordance with the findings of Sato et al [6], who found improvement in integral-based VEM identification over MLR, but still obtained negative parameter values. The IM failure to consistently produce accurate parameter values might be due to un-modeled effects in the *p*_*aw*_*(t)* term and the subsequent amplification of error via the comparatively highly parameterized model. Less parameterized models have been robust to such estimation of the simulated profile and successfully identified with the IM [19,26,27].

In contrast to the IM, the IIM enabled successful parameter identification in every case tested. Table 2 highlights a case wherein the IM (effectively the first cycle of IIM) yielded a highly un-physiological value for *C*_*2*_. In this case, the IIM converged to a lower error state with more plausible physiological values. By repeatedly evaluating the matrix equation Eq. (12) the *p*_*aw*_*(t)* term in the left hand side is updated, whereas the *p*_*aw*_*(t)* on the right hand side remains the measured data. Thus, the linear least square error minimization process optimizes the simulated *p*_*aw*_*(t)* against the measured values directly. Figure 5 shows that the IIM and the SSM located the same parameter values and Table 1 shows that the methods found the lowest overall error values.

The LMA is a popular parameter identification method for linear and non-linear problems [9,28,29]. LMA parameter identification in clinical data was 3.5 times faster than the SSM due to its higher order, but still 14 times slower than the IIM. However, the LMA appears more sensitive to initial values than the SSM. With the same hierarchically derived initial values, the LMA reported local minima with comparatively high SSE values in 9 out of 26 (35%) data sets. Based on this study, the LMA with hierarchical support is not suitable for reliable straightforward VEM parameter identification.

The IIM and SSM located very similar minimal error in every case. In contrast, MLR consistently failed in the presence of measurement error and noise. The IM was unable to report the error minima located by IIM and SSM, perhaps due to un-modeled effects in *p*_*aw*_. LMA occasionally converged successfully, but located local minima in a significant number of cases. Hence, only the IIM and SSM could be considered as reliable methods for VEM parameter identification. The robustness of the SSM was dependent on the initial values, which were provided here by a hierarchical method. In contrast, the IIM was independent of initial values, which is a significant reduction in complexity and is thus more robust in application.

### Limitations

All models are representations of a true system and thus all models have limitations. In this case, the underlying VEM is a basic model and is limited in its abilities to describe all effects in respiratory mechanics, in large part to capture fundamental physiology and also to ensure it is identifiable with readily available data or simple maneuvers. Pathophysiological effects that are not captured by the VEM include for example alveolar recruitment and over-distension, flow limitation, and edema among leading issues that can affect such models and experiments. Assuming these un-modeled effects are not present in the data, a perfect model agreement could be guaranteed, given a perfect experiment.

However, additional un-controlled effects are always present in clinical data due to measurement noise and the clinical impact of these devices on physiological responses. Secondly, inadequate experimental handling e.g. spontaneous muscle activities caused by insufficient sedation interferes with the assumed experimental hypotheses and model assumptions that neglect this activity. With this variety of un-modeled effects in the data, the IM can report inaccurate results that could be corrected by applying the IIM.

For example, assuming a hypothetical impulse of pressure in the measured data at *t = t*_*2*_ due to any of the above described reasons, then the integral of the pressure will be affected from *t*_*2*_ to *t*_*end*_ in Eq 8. Hence, the IM will have the aberration in both sides of the equation and the model functions that are most prominent in the period *t*_*2*_ will be negatively affected. In contrast, the IIM uses a re-simulated model response and thus, the impulse gets smoothed in the left hand side of Eq 8 over one or more iterations. The error induced by the impulse at *t = t*_*2*_ is present in the model simulation contained in the left hand side of Eq 8 from *t = 0* to *t*_*end*_. The error is not limited to a particular period of the response and thus the error does not exaggerate any particular model function. With ongoing iterations the influence of the impulse is reduced, and the results of the IIM approach the solution of the gradient-based methods. Hence, the IIM offers built-in model-based compensation for such errors within its framework, as the iterations enforce the model dynamics onto the data mitigating the impact of un-modeled effects. These effects are presumed small, although it should be noted that this presumes the model itself captures the fundamental mechanics accurately.

Small deviations within the reported parameter values of both methods were found (Figure 5). These deviations are well within clinical tolerances and were unlikely to significantly alter therapeutic choices. The lowest correlation (R = 0.986) of the IIM parameters to the SSM parameters were found in *R*_*2*_, which is, together with *C*_*2*_*,* predominantly sensitive to the relaxation phase [30]. This phase is most-likely to be disturbed by cardiogenic oscillations. Additionally, in cases with no distinct exponential pressure drops during EIP, information content for *R*_*2*_*, C*_*2*_ identification is low, decreasing the convexity in the error plane. Low convexity causes an early termination of the tested identification algorithms leading to minor deviation in the corresponding parameters. Changes in tolerances used would also improve these small differences but are with a variation of less than 1% utilizing a TolFun value being 10 times smaller, relatively small.

### Technical and clinical impact

The IIM is potentially applicable to other linear respiratory mechanics models of higher order, such as the Inhomogeneity Model [31], as this model poses a transfer function of the same form as the VEM [32]. Furthermore, the advantage of the IIM of being independent on initial values is beneficial for the principle of hierarchical approaches of parameter identification. A robust identification process of more advanced models like the VEM avoids gradient-based methods within this hierarchical level and shifts these more susceptible methods further down in the model hierarchy. Thus, an implementation of IIM would lead to increased robustness and efficiency in hierarchical approaches of more extensive hierarchical model structures [33].

Applying the IIM to the VEM allows online-monitoring the trends of patient-specific viscoelasticity at the bedside, and thus supports controlling the mechanical ventilator to maintain the viscoelastic properties in a normal range. Secondly, using a frequency dependent model supports the adjustment of ventilation frequency at the lowest impedance. Further clinical studies are required to develop a control scheme based on viscoelastic properties of the individual patient’s lung.

## Conclusion

Compared to gradient-based methods, the IIM provides robust parameter identification and operates without the initial value problem. Therefore it can offer more operator independence in model parameter identification.

The VEM investigations confirm that parameter identification via the IIM is also superior to error-mapping methods with respect to efficiency and robustness. The IIM takes advantage of the linear mathematical structure of the model and offers fast computing time and maximal robustness. These features offer potential for the IIM to be implemented in an online tool at the bedside for ventilation management.

## Abbreviations

ARDS, Acute Respiratory Distress Syndrome; C, Compliance in FOM; C1, Static compliance in VEM; C2, Viscoelastic compliance in VEM; EIP, End-inspiratory pause; FOM, 1st Order Model; ICU, Intensive Care Unit; IIM, Iterative Integral Method; IM, Integral Method; LMA, Levenberg-Marquardt Algorithm; LSE, Least Square Error; MLR, Multiple linear regression; MV, Mechanical Ventilation; paw, Airway pressure; pC1, Pressure in C1; pC2, Pressure in C2; R, Resistance in FOM; R1, Airway resistance in VEM; R2, Viscoelastic resistance in VEM; SSE, Sum of squared errors; SSM, Simplex-Search Method; TolFun, Terminating Function Tolerance; TolX, Terminating Parameter Tolerance; VCV, Volume-Controlled-Ventilation; VEM, Viscoelastic Model; XVEM, Unknown patient-specific VEM parameter; τ, Time constant; V˙, Flow rate.

## Competing interest

The authors declare that they have no competing interests.

## Authors’ contributions

CS, PDD, YSC, JGC developed the methods for this particular model and created the experimental setups. JGC and KM had input to analysis of the results. All authors had input in writing and revising the manuscript.
